# An Integrative Approach to Identifying Neuroprotective Natural Compounds for Neurodevelopmental Disorders

**DOI:** 10.3390/ijms26188873

**Published:** 2025-09-12

**Authors:** Juliana Alves da Costa Ribeiro Souza, Rafael Martins Xavier, Terezinha Souza, Davi Farias

**Affiliations:** 1 Postgraduate Program in Bioactive Natural and Synthetic Products, Federal University of Paraíba, Joao Pessoa 58051-970, Paraiba, Brazil; julianaacrs@hotmail.com; 2 Laboratory for Risk Assessment of Novel Technologies, Department of Molecular Biology, Federal University of Paraiba, Joao Pessoa 58051-900, Paraiba, Brazil; rafaelcbiotec@gmail.com (R.M.X.); terezinhamsouza@gmail.com (T.S.)

**Keywords:** drug discovery, computational pharmacology, natural compounds, neurological disorders

## Abstract

Neurodevelopmental disorders (NDDs) represent significant public health challenges due to their multifactorial etiology and clinical heterogeneity. Current treatments remain limited, highlighting the need for novel therapeutic strategies. This study aimed to identify neuroprotective natural compounds targeting NDD-associated pathways and describe an integrative computational pipeline combining in silico screening, network pharmacology, and molecular docking approaches to accelerate NDD drug discovery. An integrative computational pipeline was developed through sequential phases: (1) systematic screening of the Traditional Chinese Medicine Systems Pharmacology Database (TCMSP) for natural compounds meeting drug-likeness criteria and toxicity thresholds; (2) biological activity prediction; (3) network pharmacology analysis integrating compound targets and NDD-associated genes; (4) protein–protein interaction network construction and functional enrichment; and (5) molecular docking validation of top compounds against prioritized targets. From 2634 initial compounds, 10 met all selection criteria. Network analysis revealed significant interactions between compound targets and NDD-associated genes, with enrichment in neurodevelopment, cognition, and synaptic regulation pathways. Three key targets emerged as hubs: CSNK2B, GRIN1, and MAPK1. Molecular docking demonstrated high-affinity binding of caryophyllene oxide, linoleic acid, and tangeretin, supported by stable interactions with catalytic residues. This study identifies caryophyllene oxide, linoleic acid, and tangeretin as promising multi-target compounds for NDD intervention, with verified interactions against key neurodevelopmental targets. The integrative computational pipeline effectively bridges traditional medicine knowledge with modern drug discovery, offering a strategy to accelerate neurotherapeutic development while reducing experimental costs. These findings warrant further experimental validation of the prioritized compounds.

## 1. Introduction

Neurodevelopmental disorders (NDDs) represent a heterogeneous group of conditions originating from atypical alterations in brain development, primarily occurring during the prenatal stages and typically becoming apparent in childhood or adolescence. Although phenotypically variable, these disorders exhibit core symptomatic overlaps, including cognitive deficits, motor dysfunction, language impairments, and emotional regulation difficulties [1,2].

The Diagnostic and Statistical Manual of Mental Disorders (DSM-5) classifies NDDs as encompassing Intellectual Disability, Communication Disorders, Autism Spectrum Disorder (ASD), Attention-Deficit/Hyperactivity Disorder (ADHD), Neurodevelopmental Motor Disorders, and Specific Learning Disorders [3]. While demonstrating strong heritability, these conditions emerge through complex gene–environment interactions that influence neurodevelopmental trajectories [2,4].

Evaluating genetic risk in NDDs is challenging due to their genetic heterogeneity. ASD exemplifies this complexity, with hundreds of implicated risk genes showing minimal overlap between affected individuals [5,6]. Compounding this genetic variability, adverse environmental exposures interact with susceptibility loci to disrupt typical neurodevelopment, underscoring the critical role of gene–environment interplay in the pathophysiological mechanisms of NDDs [7].

NDDs pose an escalating global health challenge, significantly compromising quality of life and creating substantial socioeconomic impacts on healthcare systems. Although pharmacological treatments have advanced, the need for novel therapeutic approaches remains evident [8]. The clinical heterogeneity of NDDs and the intricate gene–environment dynamics render single-gene targeting strategies particularly challenging and with limited therapeutic scope. Consequently, pathway-based approaches that modulate core neurodevelopmental mechanisms may offer more robust and comprehensive treatment alternatives [9,10].

Building on these pathway-based therapeutic opportunities, mechanistic studies consistently highlight three pathophysiological domains: (I) protein synthesis, (II) epigenetic transcriptional regulation, and (III) synaptic signaling [5]. In ASD specifically, dysregulated pathways with therapeutic potential include Wnt/β-catenin, Sonic Hedgehog (Shh), ERK/MAPK, and PI3K/AKT signaling, along with neuroinflammatory mediators (TGFs and JAK/STAT) and neurotransmitter systems including glutamatergic and GABAergic imbalance [11]. This pathway-level understanding enables targeted therapeutic development that addresses the multifactorial nature of these disorders.

Drug development is a lengthy and costly process, often marked by high failure rates, frequently due to efficacy or safety issues identified during clinical trials [12]. To address these challenges, computational approaches have been increasingly adopted to streamline early discovery phases and improve candidate selection. Collectively termed Computer-Aided Drug Design (CADD), these methodologies leverage in silico techniques to prioritize compounds with optimal pharmacological profiles [13].

CADD encompasses two primary approaches: Structure-Based Drug Design (SBDD) and Ligand-Based Drug Design (LBDD) [14]. SBDD utilizes computational techniques including molecular docking and molecular dynamics simulations to predict ligand–target interactions. LBDD employs Quantitative Structure–Activity Relationship (QSAR) and pharmacophore modeling to identify novel bioactive compounds based on established structure–activity patterns. Furthermore, CADD integrates predictive pharmacokinetic and toxicological profiling before experimental testing, thereby reducing costs and risks in drug development [15].

Network pharmacology represents another powerful computational drug discovery methodology that adopts a systems perspective [16]. This paradigm shift, first formalized by Hopkins [17,18], analyzes drug actions through interconnected target–pathway networks rather than single targets, enabling both drug repositioning and novel compound discovery, through polypharmacology mechanisms. The methodology has demonstrated broad therapeutic utility, with successful applications spanning cardiovascular (pulmonary hypertension), metabolic (diabetes mellitus), neurodegenerative (Alzheimer’s disease), and oncological (cancer) disorders [19,20,21,22].

The pursuit for innovative treatments for neurodevelopmental disorders has increasingly focused on natural compounds exhibiting neuroprotective properties. Notably, several of these phytochemicals exhibit antioxidant, anti-inflammatory, and neurotransmitter pathway-modulating activities, positioning them as candidates for addressing the complex pathophysiology of NDDs [23].

Among these neuroprotective phytochemicals, luteolin demonstrates CNS immunomodulation by inhibiting immune cell activation in the central nervous system and suppressing the expression of pro-inflammatory mediators such as IL-6 and TNF-α, in addition to modulating the NF-κB signaling pathway [24]. Resveratrol exerts neuroprotective effects by stimulating mitochondrial biogenesis via the SIRT1/PGC-1α pathway, thereby reducing oxidative stress and neuroinflammation [25]. Furthermore, curcumin exhibits antioxidant and anti-inflammatory properties, improving intracellular glutathione levels, reducing the production of reactive oxygen species (ROS), and protecting against mitochondrial damage, in addition to modulating inflammatory factors involved in the pathophysiology of neurodevelopmental disorders [26].

Given the demonstrated ability of natural compounds to modulate key biological pathways in NDDs, there is a pressing need to systematically identify novel neuroprotective molecules [27]. While current pharmacotherapy primarily addresses symptoms (e.g., stimulants for ADHD, risperidone for irritability in ASD), natural compounds offer potential disease-modifying effects by targeting underlying mechanisms [28]. The discovery of such molecules could enable the development of (1) complementary approaches to enhance existing therapies, (2) multi-target strategies addressing NDD complexity, and (3) personalized interventions based on individual pathway dysregulation.

This study aims to identify novel neuroprotective natural compounds for NDDs through an integrated computational approach combining in silico screening and network pharmacology. The innovative methodology employs a phased screening strategy that systematically evaluates pharmacokinetic properties, toxicity profiles, biological activity predictions, and mechanism-of-action analyses.

This multi-parametric approach enables more accurate drug candidate selection, optimizing the identification of promising compounds while significantly reducing the time and costs associated with early-stage drug development. By integrating critical pharmacological parameters into a unified pipeline, our strategy overcomes limitations of conventional drug discovery methods, offering an efficient pathway for developing multi-target therapies directed at core NDD pathophysiological mechanisms.

## 2. Results

**Figure 1:**
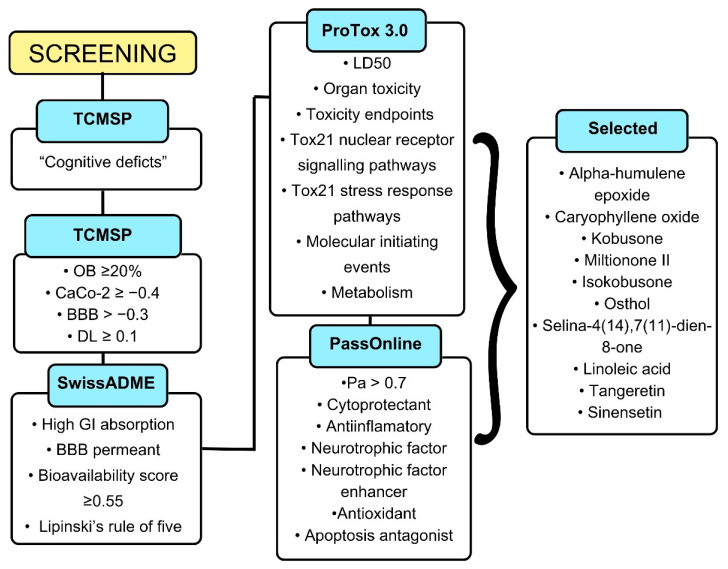
Flowchart of screening process for natural compounds with neuroprotective potential.

**Figure 2:**
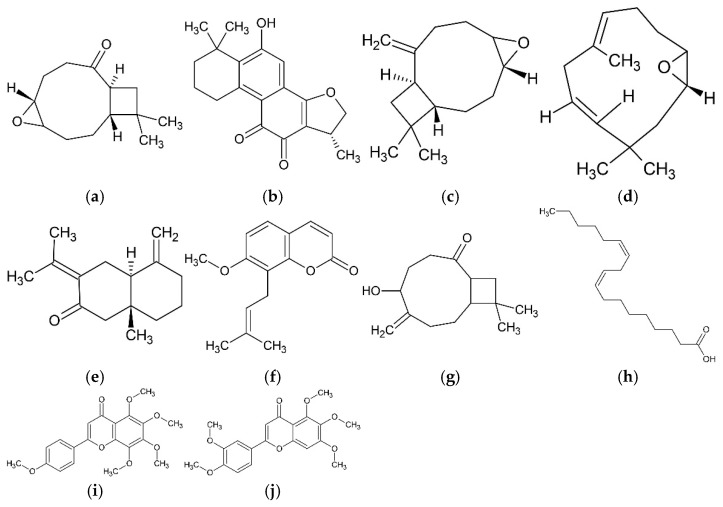
The chemical structures of the selected natural compounds: (**a**) kobusone, (**b**) miltionone II, (**c**) caryophyllene oxide, (**d**) α-humulene epoxide, (**e**) selina-4(14),7(11)-dien-8-one, (**f**) osthol, (**g**) isokobusone, (**h**) linoleic acid, (**i**) tangeretin, and (**j**) sinensetin.

The natural compounds were systematically evaluated for key pharmacological properties, including drug-likeness and blood–brain barrier (BBB) permeability potential, followed by comprehensive toxicity endpoint and biological activity predictions (Figure 1). Through this multi-stage screening pipeline, ten compounds demonstrating favorable pharmacological profiles were selected for further investigation. The final candidates comprised six terpenoids [kobusone, caryophyllene oxide, α-humulene epoxide, isokobusone, selina-4(14),7(11)-dien-8-one, and miltionone II], one coumarin (osthol), two flavonoids (tangeretin and sinensetin), and one fatty acid (linoleic acid) (Figure 2).

The results generated from the screening of these selected compounds are detailed in the following sections.

### 2.1. Compound Screening and Selection

An initial pool of 2634 natural compounds was retrieved from the TCMSP database using ‘cognitive deficits’ as the primary search criterion. Sequential filtering through established drug-likeness parameters (TCMSP and SwissADME criteria) and removal of duplicate entries yielded 460 qualified candidates. The rigorous filtration process reduced the initial dataset by 82.5%, focusing on compounds with clinically translatable potential for cognitive enhancement. The parameter values evaluated for the ten selected natural compounds are presented in Table 1

### 2.2. Comprehensive Toxicity Evaluation

**Figure 3:**
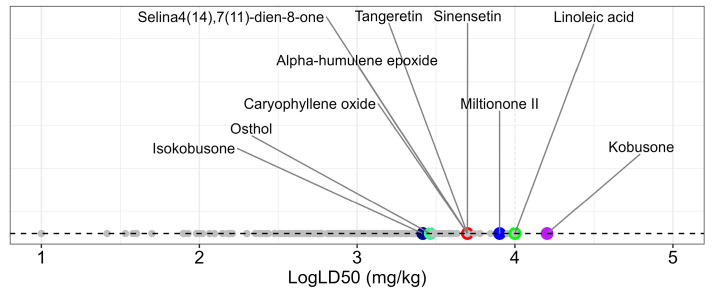
The predicted LD50 values of the natural compounds on a logarithmic scale. The dots represent the log10(LD50) values for each compound analyzed. Selected compounds are indicated with specific colors and associated names. Smaller gray dots correspond to non-selected compounds.

The canonical SMILES representations of the 460 compounds were submitted to the ProTox 3.0 platform for systematic toxicity evaluation. The predictions spanned the complete toxicity classification spectrum (Class I to VI), with LD50 values ranging from 1 to 16,000 mg/kg. To enhance data visualization, the LD50 values were log-transformed and are presented in Figure 3, revealing clear toxicity gradients among the evaluated compounds.

Among the 10 selected compounds, kobusone exhibited the highest predicted safety margin (LD50 = 16,000 mg/kg), while isokobusone showed the lowest values (LD50 = 2610 mg/kg). Based on the ProTox 3.0 classifications, 113 compounds meeting the stringent Class V and VI safety criteria were advanced for subsequent toxicity modeling analyses. 

The implemented toxicity screening protocol successfully identified compounds with favorable safety profiles suitable for further pharmacological investigation. This approach not only excluded potentially hazardous compounds but also established a quantitative framework for comparing toxicity profiles across different chemical classes.

**Figure 4:**
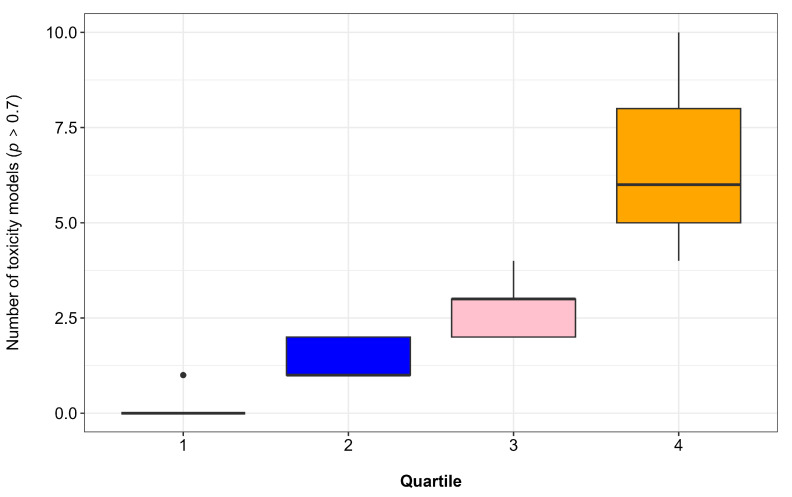
Classification of compounds into quartiles based on the number of endpoints with a toxicity probability ≥ 0.7. Each color represents a quartile, allowing for visualization of compounds with lower toxicity levels (Q1) compared to the most toxic ones (Q4). The dot above Quartile 1 represents a compound with one predicted toxic endpoint (*p* > 0.7).

The compounds were systematically assessed within the ProTox 3.0 platform through multiple toxicity prediction models, including organ toxicity, toxicity endpoints, Tox21 nuclear receptor pathways, Tox21 stress response pathways, and metabolic toxicity. For each compound, the number of toxicity models yielding probabilities ≥ 0.7 was quantified, along with the cumulative sum of these probabilities. This dual-metric approach enabled robust toxicity profiling and subsequent quartile-based classification of all compounds, as visually represented in Figure 4.

Following quartile stratification, compounds within the fourth quartile (Q4), representing those with the highest toxicity potential, were systematically excluded from further analysis. This stringent filtering process resulted in the selection of 84 compounds with more favorable toxicity profiles for subsequent biological activity prediction. The quartile-based elimination approach ensured that only compounds demonstrating lower probabilities of toxicity across multiple endpoints were advanced in the screening pipeline, thereby increasing the likelihood of identifying safe and potentially bioactive candidates.

### 2.3. Biological Activity Prediction

**Figure 5:**
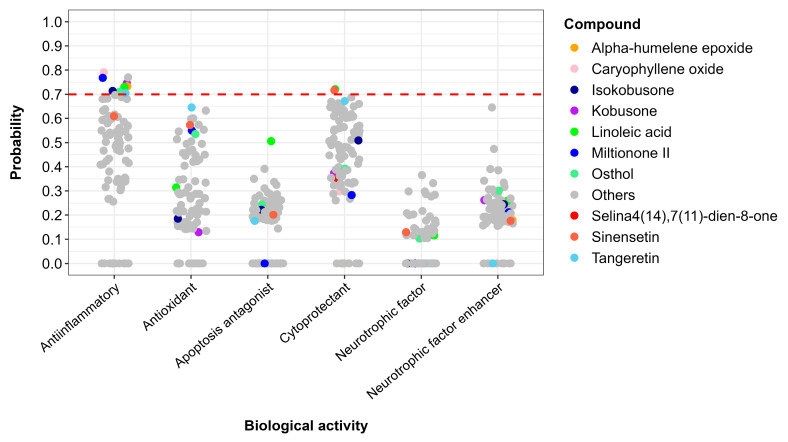
Predicted probabilities from the PASS online database for biological activities related to the neuroprotection of natural compounds, with the most promising compounds highlighted in specific colors. A probability cutoff of 0.7 is marked by the dashed red line.

Compounds demonstrating favorable toxicity profiles were further evaluated for their potential biological activities using the PASS online prediction platform. Based on stringent selection criteria (probability > 0.7), ten compounds were identified as having a high likelihood of exhibiting pharmacologically relevant activities. Among these, all compounds except sinensetin showed significant potential for anti-inflammatory effects. Additionally, both sinensetin and linoleic acid exhibited a high probability of cytoprotective activity, as illustrated in Figure 5.

### 2.4. Target Prediction and PPI Network Construction of Compounds and NDDs

Molecular targets associated with neurodevelopmental disorders were systematically retrieved from the OMIM and GeneCards databases using the search terms “neurodevelopmental disorder” and “neurodevelopmental abnormalities.” Following data standardization in UniProt and removal of duplicate entries, 4671 unique targets were identified. In parallel, potential targets of the natural compounds were predicted using the SwissTargetPrediction and SuperPred platforms, which identified between 146 and 228 targets per compound. Among these, sinensetin (221 targets) and tangeretin (220 targets) exhibited the most extensive target profiles.

**Figure 6:**
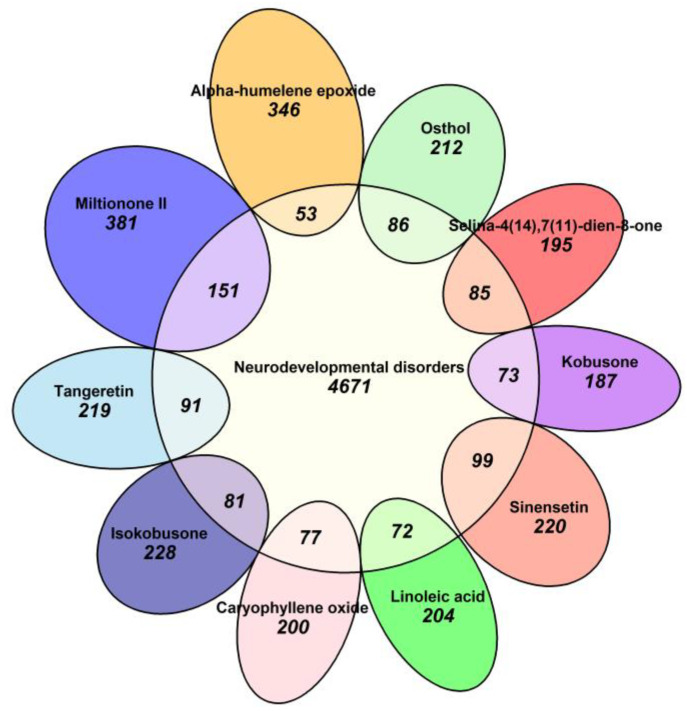
A Euler diagram showing the overlap between molecular targets of natural compounds and targets associated with neurodevelopmental disorders. The internal numbers indicate the quantity of shared targets between each compound and neurodevelopmental disorder targets, while external numbers represent the count of unique targets.

The intersection between compound targets and neurodevelopmental disorder-related targets was evaluated using a Venn diagram approach. This analysis revealed significant overlaps for tangeretin (91 shared targets), sinensetin (99 shared targets), and selina-4(14),7(11)-dien-8-one (85 shared targets). These interactions were further visualized using a Euler diagram (Figure 6), which provided a comprehensive representation of the compound–target networks and their shared associations with neurodevelopmental disorders.

### 2.5. Enriched Pathways

The selected natural compounds were evaluated for their ability to modulate 15 neurodevelopmentally relevant pathways spanning three major biological annotation systems: Gene Ontology Biological Processes, Reactome Pathway database, and Monarch Disease Ontology. This comprehensive analysis revealed significant associations between multiple compounds and pathways governing fundamental neurodevelopmental processes, including neurogenesis regulation, synaptic plasticity, and cognitive function. The findings demonstrate the compounds’ potential to influence key molecular events underlying neurodevelopmental disorder pathophysiology.

The terpenoid compounds selina-4(14),7(11)-dien-8-one and isokobusone showed strong associations with core neurodevelopmental biological processes, including nervous system development (GO:0007399), cognition (GO:0050890), and learning or memory (GO:0007611). The flavonoids sinensetin and tangeretin exhibited significant modulation of pathways linked to clinical neurodevelopmental phenotypes, particularly those annotated in the Monarch Disease Ontology including neurodevelopmental abnormality (HP:0012759), cognitive impairment (HP:0100543), and intellectual disability (HP:0001249). Notably, caryophyllene oxide demonstrated involvement in both molecular mechanisms (axon guidance—R-HSA-422475) and behavioral phenotypes (autistic behavior—HP:0000729), as well as showing significant association with cognition-related pathways (GO:0050890).

**Figure 7:**
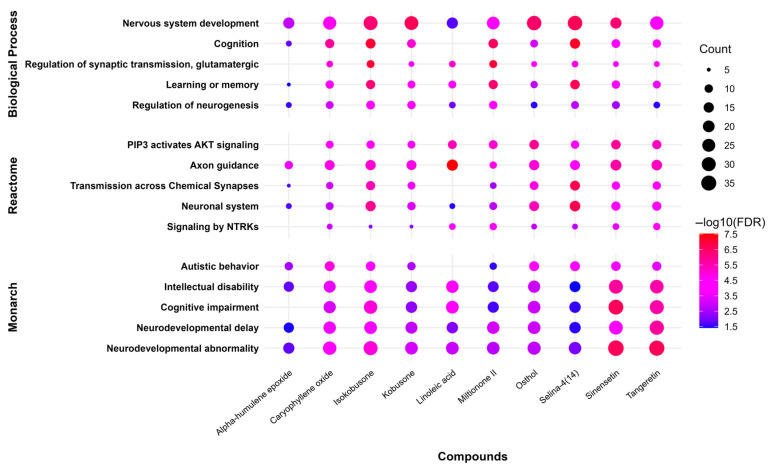
Graphical representation of selected Gene Ontology (Biological Process), Reactome Pathway, and Monarch pathways, with circle sizes representing gene counts and color gradient (blue-to-red) showing −log10(FDR) values for compound–pathway associations.

The compound–pathway interaction network is visualized through a multidimensional representation (Figure 7) employing two key analytical metrics: (1) pathway engagement breadth, represented by a circle diameter proportional to the number of associated genes, and (2) statistical significance, depicted through a red-to-blue color gradient corresponding to −log10(FDR) values. This visualization approach simultaneously communicates both the extensiveness and confidence of each compound–pathway association, with warmer hues (red spectrum) indicating more statistically robust interactions (FDR < 0.001) and larger circles representing pathways involving greater numbers of molecular targets.

### 2.6. Compound–Target–Pathway Interactions

The systematic analysis of compound–target–pathway interactions was performed using Cytoscape 3.10.3, generating an integrated network comprising 253 nodes (10 compounds, 15 pathways, and 228 targets). This network architecture was specifically designed to elucidate the complex relationships between phytochemical compounds and their potential mechanisms of action in neurodevelopmental contexts.

Using CytoHubba’s degree centrality algorithm, three critical hub targets were identified: CSNK2B (degree = 18), GRIN1 (degree = 16), and MAPK1 (degree = 16). These targets occupied central positions in the network, serving as major connection points between multiple compounds and pathways. Concurrently, five pathways demonstrated exceptional connectivity: neurodevelopmental abnormality (degree = 97), nervous system development (degree = 96), cognitive impairment (degree = 87), intellectual disability (degree = 81), and neurodevelopmental delay (degree = 81). The high degree values of these elements suggest their fundamental role in the predicted pharmacological effects.

**Figure 8:**
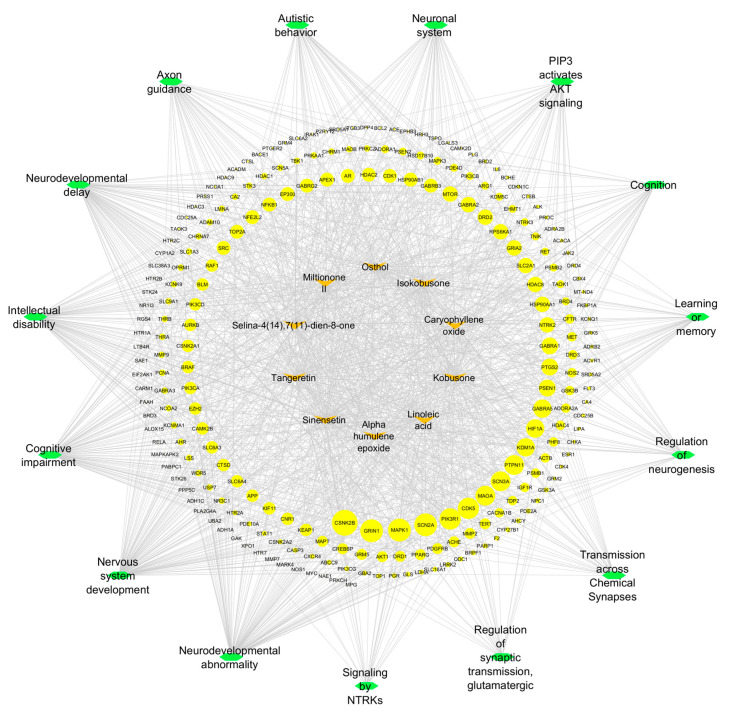
The relationships between the selected natural compounds (central green nodes), enriched pathways (peripheral orange nodes), and molecular targets (yellow nodes). Node size scales proportionally with degree centrality, reflecting each element’s connectivity within the network.

The network visualization (Figure 8) employs a hierarchical layout with three distinct topological layers: (1) an outer ring containing the natural compounds, (2) an intermediate layer of target proteins (blue nodes, size-scaled by degree centrality), and (3) a central cluster of pathways (green nodes).

### 2.7. Analysis of Molecular Docking Interactions

A comprehensive molecular docking analysis was conducted to characterize the binding interactions between selected natural compounds and three pivotal protein targets implicated in neurodevelopmental disorders: CSNK2B (PDB ID 3EED), GRIN1 (PDB ID 5H8H), and MAPK1 (PDB ID 6SLG). The study evaluated binding affinities, specific residue interactions, and intermolecular distances to assess potential therapeutic applications.

For CSNK2B, caryophyllene oxide exhibited the most favorable binding energy (−5.4 kcal/mol), though its interaction with ARG150 occurred at suboptimal distances. Tangeretin demonstrated a more stable hydrogen bond with LYS147 (−4.7 kcal/mol), suggesting alternative binding mechanisms. GRIN1 analysis revealed caryophyllene oxide’s superior affinity (−6.7 kcal/mol versus native ligand’s −5.9 kcal/mol), mediated through π-π and π-alkyl interactions with TYR144 and PRO141. Linoleic acid also showed promising binding (−5.9 kcal/mol) via similar interactions. MAPK1 docking identified caryophyllene oxide (−9.2 kcal/mol) and miltionone II (−9.1 kcal/mol) as near-native binders, primarily engaging VAL39 through π-alkyl interactions.

**Figure 9:**
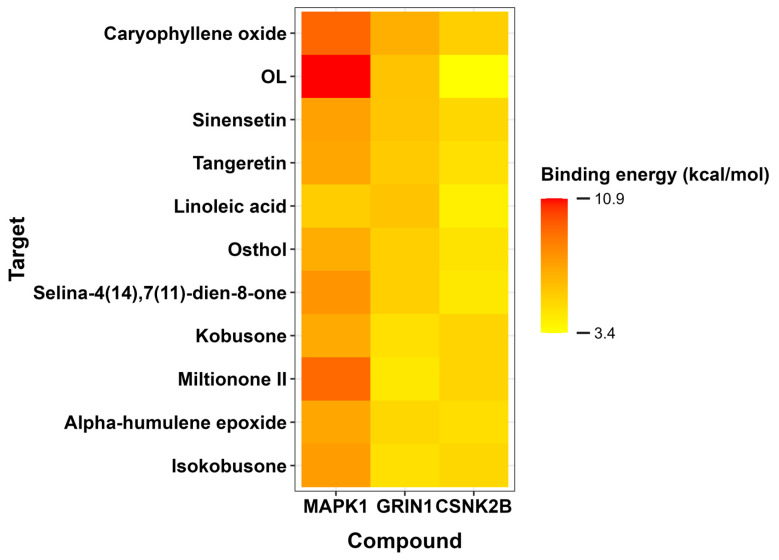
The heatmap displays the predicted binding energies of natural compounds (y-axis) against the three target proteins (x-axis). The yellow-to-red color gradient represents the binding affinity values, with red indicating the most favorable (lowest) binding energies and yellow corresponding to weaker interactions. OL denotes the original reference ligand for each protein target.

**Figure 10:**
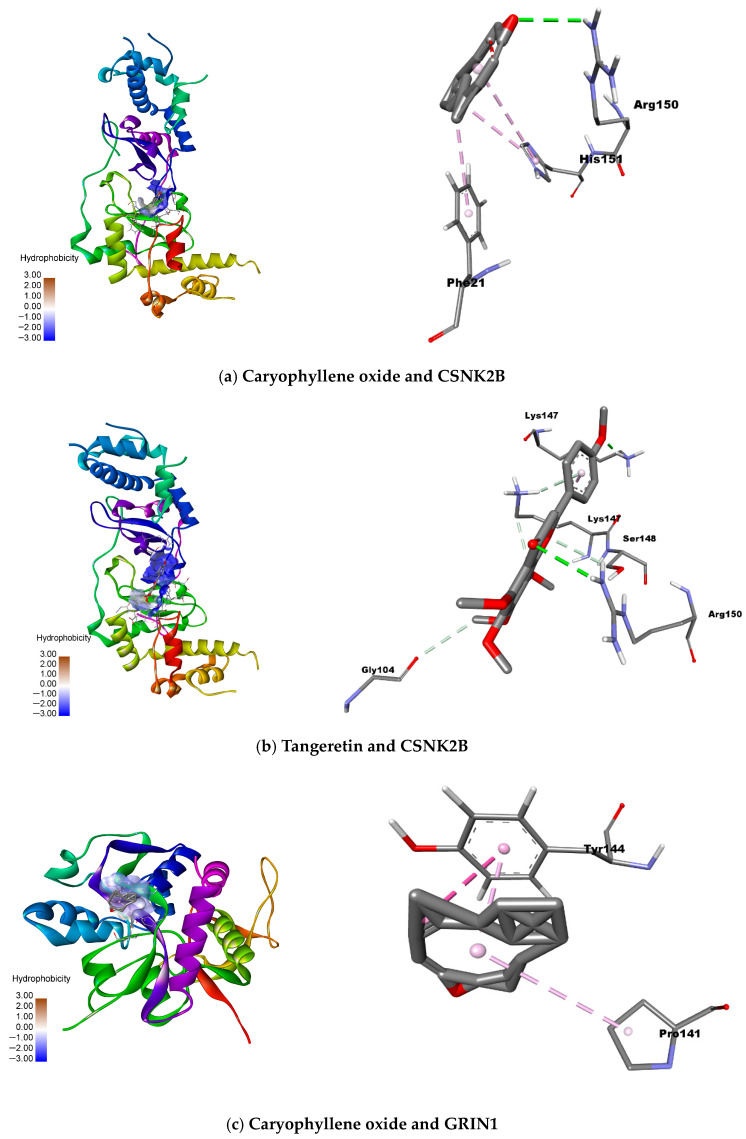

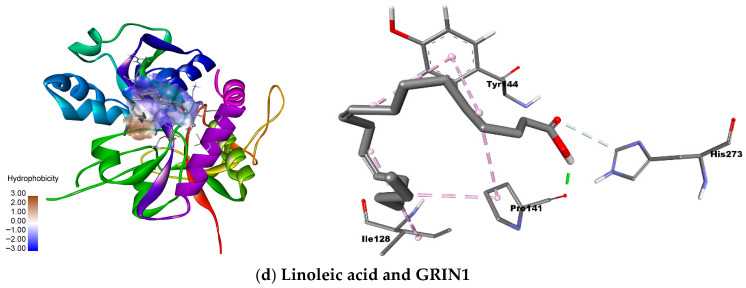
A structural representation of the most relevant binding interactions between natural compounds and target proteins: (**a**) caryophyllene oxide and CSNK2B, (**b**) tangeretin and CSNK2B, (**c**) caryophyllene oxide and GRIN1, and (**d**) linoleic acid and GRIN1.

The heatmap visualization (Figure 9) employs a yellow-to-red color gradient to differentiate compound affinities across targets, where red indicates the most favorable binding energies (lowest values) and yellow represents progressively weaker interactions. This chromatic scale clearly reveals caryophyllene oxide’s consistent strong binding (predominantly red/orange) across all three protein targets, while Figure 10 provides structural details of the specific molecular interactions. Notably, the color gradient shows caryophyllene oxide emerging as a particularly promising multi-target ligand, with its darkest red coloration (−9.2 kcal/mol with MAPK1) indicating exceptional binding affinity approaching that of the original ligands.

## 3. Discussion

Computer-Aided Drug Design (CADD) has emerged as a transformative approach in modern drug discovery, enabling the rational and computationally driven optimization of lead compounds [29,30]. The present study employed an integrated CADD workflow that synergistically combined ligand-based and structure-based strategies. The implemented screening protocol adopted a rigorous, multi-stage filtering process beginning with an initial library of 2634 natural compounds. The pipeline incorporated successive evaluation gates including (1) drug-likeness assessment, (2) blood–brain barrier permeability, (3) comprehensive toxicity profiling through ProTox 3.0 and quartile-based classification, and (4) biological activity prediction focusing on neuroprotective mechanisms.

Concurrently, structure-based methods, including molecular docking and network pharmacology, were employed to validate target engagement and polypharmacological potential. This hierarchical strategy achieved a remarkable 99.6% reduction in candidate compounds while preserving those with optimal multi-target profiles, such as caryophyllene oxide, which demonstrated both high predicted biological activity and favorable binding affinities across all three key targets (CSNK2B, GRIN1, and MAPK1).

The demonstrated effectiveness of this computational pipeline holds particular significance for central nervous system drug development, where the blood–brain barrier presents a major obstacle and polypharmacology is often desirable [31]. As inadequate ADMET properties remain a primary cause of clinical-phase failures [32], the current methodology offers a comprehensive solution by combining biological activity prediction, target validation, and ADMET profiling within a unified workflow. These results suggest that such computational frameworks can significantly accelerate the discovery of novel neurotherapeutics while reducing development costs and timelines [33].

The molecular docking analysis identified significant interactions between selected natural compounds and key neuronal targets, with particularly strong binding affinities observed for caryophyllene oxide and tangeretin with CSNK2B (−5.4 and −4.7 kcal/mol, respectively), and for caryophyllene oxide and linoleic acid with GRIN1 (−6.7 and −5.9 kcal/mol). These results suggest that these compounds may effectively modulate critical neuronal pathways through specific protein interactions, as confirmed by their favorable binding energies and interaction patterns with key catalytic residues.

The CSNK2B gene encodes the β subunit of casein kinase 2 (CK2), a constitutively active serine/threonine kinase that plays fundamental roles in neuronal morphology and synaptic transmission [34]. CK2 participates in multiple essential nervous system processes, including neuronal migration, cell adhesion, synaptic plasticity, and modulation of neurotransmitter receptors such as ion channels and G protein-coupled receptors (GPCRs) [35]. This ubiquitous kinase regulates diverse cellular signaling pathways through its pleiotropic phosphorylation activity, positioning it as a crucial regulator of neuronal function and development.

CK2 orchestrates several key cellular signaling cascades that are particularly relevant to neuronal survival and function. The kinase positively regulates the Akt/GSK3β pathway, which is essential for cell growth and survival, while simultaneously inhibiting PTEN to prevent negative regulation of the PI3K/Akt pathway [36]. Additionally, CK2 modulates pro-inflammatory pathways through phosphorylation of IκBα in the NF-κB pathway, promoting its degradation and subsequent NF-κB release. CK2 also amplifies cytokine signaling through the JAK2/STAT3 pathway, demonstrating its broad involvement in both neuronal and immune regulation [37].

Aberrant CK2 activity has been implicated in various neurodevelopmental and neurological disorders. In neurodevelopmental conditions, CK2 dysfunction is associated with ASD [35], Okur–Chung (OCNDS) and Poirier–Bienvenu (POBINDS) syndromes [36], and Attention-Deficit Hyperactivity Disorder [37]. Furthermore, CK2 alterations are involved in several neurodegenerative and neurological diseases, including Alzheimer’s disease (AD), Parkinson’s disease (PD), amyotrophic lateral sclerosis, multiple sclerosis, and epilepsy [34]. These widespread associations highlight CK2’s central role in maintaining neuronal homeostasis.

Current research positions CK2 as a promising therapeutic target for epilepsy, with evidence showing that its inhibition reduces aberrant neuronal activity and seizures [38]. For AD, PD, ASD, and multiple sclerosis, CK2 is considered a potential therapeutic target, although no clinical treatments have yet been approved [39,40]. A major challenge in developing CK2-targeted neurological therapies is the poor blood–brain barrier (BBB) permeability of current selective inhibitors such as DMAT, TBB, and CX-4945 [41]. This limitation underscores the importance of identifying natural compounds with both CK2-modulating activity and favorable BBB penetration properties for developing effective neurological therapies.

The GRIN1 gene encodes the GluN1 subunit of N-methyl-D-aspartate receptors (NMDARs), which are ubiquitously expressed throughout the central nervous system. These receptors play pivotal roles in synaptic plasticity and neuronal development, fundamental processes underlying cognitive function. NMDAR activity must be precisely regulated within a narrow physiological range, as both excessive and insufficient activity can disrupt glutamatergic synapses and compromise neuronal integrity [42]. This delicate balance underscores the critical importance of proper GRIN1 function in maintaining normal brain development and function. Numerous studies have linked GRIN1 mutations to various neurodevelopmental disorders, including ASD, epilepsy, schizophrenia, and intellectual disability [43].

The GluN1 subunit represents a particularly promising therapeutic target for neurodevelopmental disorders due to its obligatory role in NMDAR assembly and function [44]. Pharmacological modulation of receptors containing GluN1 subunits offers unique opportunities to fine-tune synaptic responses and potentially correct imbalances in glutamatergic neurotransmission. This approach could address both NMDAR hypofunction and hyperactivity, which have been implicated in various neurodevelopmental conditions [45].

The development of compounds that selectively target GluN1-containing receptors while preserving physiological NMDAR activity remains an active area of investigation. Despite the therapeutic potential of GluN1-targeted approaches, significant challenges remain in developing safe and effective treatments. A major obstacle involves achieving sufficient subunit selectivity to avoid disrupting normal receptor function in unaffected neural circuits [46]. The identification of natural compounds with favorable binding properties to the GluN1 subunit, as demonstrated in our molecular docking studies, may provide new avenues for developing safer and more effective treatments for GRIN1-related neurodevelopmental disorders.

NDDs represent a significant therapeutic challenge due to their multifactorial etiology and the side effects associated with conventional pharmacological treatments [47]. While current medications can effectively alleviate symptoms, their primary focus on symptomatic relief highlights the need for developing safer and more efficient therapies capable of potentially preventing or reversing neurological dysfunction [23]. This pressing medical need has driven increasing interest in alternative therapeutic approaches that address the complex pathophysiology underlying these disorders.

In the present study, three natural compounds—caryophyllene oxide (BCPO), tangeretin (TG), and linoleic acid (LA)—emerged as particularly promising candidates for NDD therapy. These compounds demonstrated not only significant molecular interactions with key targets (CSNK2B, GRIN1, and MAPK1) but also a high probability of modulating pathways relevant to neurodevelopmental processes. Their multi-target activity profile suggests potential advantages over conventional single-target pharmacological approaches for addressing the complex neurobiology of developmental disorders.

BCPO, a major sesquiterpenoid found in various essential oils, has attracted considerable scientific interest due to its broad pharmacological potential [48]. The compound exhibits notable analgesic and anti-inflammatory effects through modulation of central pain receptors and a reduction in inflammatory mediators, suggesting its potential for treating chronic pain and neural inflammation [49]. Furthermore, BCPO has demonstrated neuroprotective properties associated with cholinesterase inhibition and antioxidant effects, which may have important implications for treating neurodegenerative diseases such as Alzheimer’s and Parkinson’s [50].

Beyond its anti-inflammatory and neuroprotective effects, BCPO has shown sedative activity in murine studies, although this effect appears to occur through mechanisms independent of GABAergic receptor modulation [51]. The compound has also demonstrated significant potential in modulating alcohol-related behaviors, showing efficacy up to ten times greater than its precursor compound β-caryophyllene in reducing ethanol intake and preference in mouse models [52]. This effect appears largely attributable to BCPO’s anti-inflammatory action against alcohol-induced neuroinflammation, further supporting its potential for modulating neurochemical pathways relevant to NDDs.

Linoleic acid is an essential polyunsaturated fatty acid abundantly found in vegetable oils such as soybean, corn, and sunflower. As a metabolic precursor, LA can be converted to γ-linolenic acid and subsequently to arachidonic acid, which plays a fundamental role in the synthesis of bioactive eicosanoids including prostaglandins and leukotrienes [53]. These metabolites are crucial for regulating key physiological processes such as inflammation, immune response, and vascular function [54], establishing LA as an important modulator of cellular signaling pathways.

LA has demonstrated significant neuroprotective properties through modulation of inflammatory responses and oxidative stress. In experimental models of Parkinson’s disease, LA exhibited antioxidant and anti-inflammatory effects, reducing neuronal damage and improving cell survival [55]. The combination of LA with zinc showed promise, preventing neuronal death and preserving midbrain integrity in rotenone-induced Parkinson’s models [56]. These findings suggest potential therapeutic applications for LA in neurodegenerative conditions, particularly through its ability to modulate multiple pathways involved in neuronal survival and function.

Despite its neuroprotective potential, emerging evidence suggests that excessive LA intake may be associated with neurodegenerative processes, primarily due to its conversion to pro-inflammatory arachidonic acid-derived eicosanoids [57]. Animal studies have shown that high LA levels can induce neuroinflammation, oxidative stress, and ataxia. Furthermore, LA metabolism in the central nervous system appears limited, with most LA being rapidly metabolized to polar products like acetate and carbon dioxide rather than being incorporated into neuronal membrane phospholipids [58]. 

The current evidence positions LA as a promising yet complex neuroprotective agent, requiring careful consideration of dosage and balance with other polyunsaturated fatty acids to maximize benefits while minimizing risks [59]. The dual nature of LA’s effects—demonstrating both neuroprotective and potentially neurotoxic properties depending on context and concentration—highlights the need for further research to establish optimal therapeutic windows and formulations. Future studies should focus on elucidating the precise mechanisms underlying these dose-dependent effects and exploring potential synergies with other neuroprotective compounds to develop safe and effective therapeutic strategies for neurological disorders.

Tangeretin, a flavonoid predominantly found in the peel of citrus fruits such as oranges and tangerines [60], has garnered increasing scientific attention due to its broad pharmacological properties and therapeutic potential. This compound exhibits a remarkable spectrum of biological activities, including antioxidant, anti-inflammatory, antitumor, anti-asthmatic, cardioprotective, and notably, neuroprotective effects [61,62], making it a promising candidate for various medical applications.

Emerging evidence positions TG as a strong candidate for treating neurodegenerative disorders such as Alzheimer’s and Parkinson’s disease. Its ability to cross the blood–brain barrier and exert neuroprotective effects through multiple mechanisms has been well documented [63]. TG modulates key cellular signaling pathways involved in neuroinflammation, including inhibition of the NF-κB and MAPK cascades, while simultaneously enhancing the expression of antioxidant factors such as superoxide dismutase (SOD) and catalase (CAT) [62,64]. These dual actions on both inflammatory and oxidative pathways contribute to its robust neuroprotective profile.

Experimental models have demonstrated that TG administration leads to significant cognitive improvement, primarily by increasing acetylcholine levels via inhibition of acetylcholinesterase activity—a mechanism shared with current Alzheimer’s medications [64]. Furthermore, TG has shown capacity to reduce neuronal apoptosis and modulate autophagy processes [65], suggesting its potential to preserve neuronal integrity and synaptic function.

Considering the demonstrated neuroprotective potential of BCPO, LA, and TG, future experimental studies should systematically evaluate safe and effective dosages through comprehensive in vitro assays (including cell viability, oxidative stress quantification, and neuroprotection-related gene expression analysis) to elucidate their mechanisms of action, followed by in vivo animal studies assessing behavioral parameters, oxidative/inflammatory biomarkers, and brain histopathology to fully characterize their therapeutic potential for neurological disorders.

## 4. Methodology

### 4.1. Pharmacokinetic Screening of Neuroprotective Natural Compounds

The initial compound library was derived from the Traditional Chinese Medicine Systems Pharmacology Database (TCMSP) version 2.3 (https://tcmsp-e.com/tcmsp.php, accessed between 1 August and 1 September 2024) using systematic search protocols. To identify molecules with potential neuroprotective activity, the platform was queried using ‘cognitive deficits’ as the primary disease descriptor. A multi-parameter pharmacokinetic filter was applied to evaluate drug-likeness: oral bioavailability (OB) ≥20%, intestinal absorption (CaCo-2 permeability) ≥ −0.4, blood–brain barrier penetration (BBB) > −0.3, and a drug-likeness score (DL) ≥ 0.1. The thresholds were selected based on TCMSP platform recommendations. 

To rigorously validate the pharmacokinetic properties of TCMSP-selected compounds, secondary screening was performed using the SwissADME platform (http://www.swissadme.ch/, accessed on 1 September 2024). The workflow involved retrieving canonical SMILES for all qualifying compounds from PubChem followed by SwissADME evaluation. The following consensus criteria were applied for CNS-penetrating compounds: (1) a bioavailability score ≥ 0.5 (optimal zone), (2) high gastrointestinal (GI) absorption probability, (3) blood–brain barrier (BBB) permeation (Boiled-Egg prediction), and (4) no more than one violation of Lipinski’s Rule of Five. Compounds satisfying both TCMSP and SwissADME criteria advanced to toxicity prediction.

### 4.2. Toxicity Prediction and Safety Profiling

To predict the toxicity of the selected compounds, their canonical SMILES were submitted to the Prediction of Toxicity of Chemicals platform (ProTox 3.0; https://tox.charite.de/protox3/, accessed between 1 October and 1 November 2024), which provides in silico toxicity predictions based on chemical structure and machine learning algorithms. ProTox-3.0 employs seven distinct predictive models: (1) acute oral toxicity (LD_50_); (2) organ toxicity; (3) toxicity endpoints; (4) Tox21 nuclear receptor pathways; (5) Tox21 stress response pathways; (6) molecular initiating events; and (7) metabolism-related toxicity.

In models 2–6, predictions are categorized based on the probability of activity (toxic) or inactivity (non-toxic), whereas in the LD_50_ model, toxicity is classified into six levels—ranging from ‘fatal if swallowed’ (Class I) to ‘non-toxic’ (Class VI). Compounds with higher LD_50_ values (class V and VI), indicating lower toxicity, were prioritized for selection. Each compound was individually evaluated for toxicity probabilities across the predictive models.

To systematically identify compounds with lower toxicity potential, we implemented a quartile-based classification approach using R statistical software (version 2024) [66]. For each compound, were calculated two key metrics: (1) the count of toxicity models with probability scores ≥ 0.7 (a well-established threshold indicating a high likelihood of toxicity [67]), and (2) the cumulative sum of these probabilities. The quartile division was performed using R’s ntile() function from the dplyr package, which automatically sorts compounds by toxicity metrics and divides them into four equal groups (each containing 25% of the dataset) based on their ranked position. This was implemented through a two-step sorting process: primary sorting by number of toxic endpoints, followed by secondary sorting (for tied compounds) using the summed probability values.

Compounds in the fourth quartile (Q4), representing the highest toxicity risk, were systematically excluded from further evaluation. The complete analytical workflow was executed in RStudio 2024 using the tidyverse ecosystem, with toxicity distributions visualized through customized boxplots that highlight the quartile stratification.

### 4.3. Prediction of Biological Activity for Natural Compounds

The canonical SMILES of natural compounds selected through toxicity screening were analyzed using the PASS Online platform 2.0 (https://www.way2drug.com/passonline/, accessed on 1 November 2024) to predict their biological activity profiles. PASS Online employs structure–activity relationship (SAR) analysis to evaluate over 4000 biological activity types, providing probability-based classifications of pharmacological activity (active/inactive).

The natural compounds were systematically screened for key neuroprotective properties, including (1) apoptosis modulation, (2) cytoprotective effects, (3) anti-inflammatory activity, (4) antioxidant capacity, and (5) neurotrophic activity [68,69]. Analysis was conducted in November 2024, using a probability threshold (Pa) ≥ 0.7 for activity prediction [67], ensuring the selection of compounds with high potential for experimental validation. This stringent cutoff was applied to all neuroprotective properties evaluated, prioritizing candidates most likely to demonstrate biological activity in subsequent assays.

### 4.4. Target Prediction for Natural Compounds and NDDs

The molecular targets of selected natural compounds were identified through the TCMSP database. Additionally, 2D compound structures were retrieved from PubChem and analyzed through both SwissTargetPrediction (http://www.swisstargetprediction.ch/, accessed on 1 November 2024) and SuperPred 3.0 (https://prediction.charite.de/, accessed on 1 November 2024), ensuring comprehensive and reliable target identification through this integrated bioinformatics strategy. Initially, compound–target associations were retrieved from the TCMSP database, which includes experimentally supported and computationally inferred targets for natural products. The 2D structures of the compounds were obtained from PubChem and submitted to each platform using default parameters.

To ensure confidence in the predicted targets, specific inclusion criteria were applied for each tool. In SwissTargetPrediction, only targets displaying a non-zero prediction probability were retained for further analysis. In SuperPred 3.0, only targets with a prediction probability greater than 50% were selected.

NDD-related targets were systematically retrieved from the GeneCards (https://www.genecards.org/, accessed on 1 November 2024) and OMIM (https://www.omim.org/, accessed on 1 November 2024). The search strategy combined the keywords “neurodevelopmental abnormalities” and “neurodevelopmental disorders” with the *Homo sapiens* species filter.

Gene nomenclature was standardized using the UniProt database (https://www.uniprot.org/, accessed on 1 November 2024) followed by removal of duplicate entries. To identify potential therapeutic targets, a comparative analysis was performed between the natural compounds’ target genes and NDD-associated genes through Venn diagram visualization using the Draw Venn Diagram tool (https://bioinformatics.psb.ugent.be/webtools/Venn/, accessed on 1 December 2024). (See Supplementary Materials for the above information).

### 4.5. PPI Network

The overlapping targets identified between natural compounds and NDD-associated genes were analyzed using STRING database 12.0 (https://string-db.org/, accessed on 1 December 2024) to construct a high confidence protein–protein interaction (PPI) network. The analysis was conducted with stringent parameters, including a minimum confidence score of 0.7 and restriction to *Homo sapiens* proteins. This network visualization represents proteins as nodes and their molecular interactions as edges, providing a systems-level understanding of potential functional relationships among the target proteins in NDDs.

### 4.6. Pathway Enrichment Analysis

Pathway enrichment analyses were conducted to elucidate the biological processes potentially modulated by the selected compounds and to prioritize the most promising neuroprotective candidates. Following construction of the protein–protein interaction (PPI) network in STRING, enrichment analyses were performed across three key biological annotation systems: (1) Biological Process terms from Gene Ontology (GO), (2) Reactome Pathway database, and (3) Monarch Initiative knowledgebase.

Pathways with a false discovery rate (FDR) < 0.01 were deemed statistically significant. To control for false positives arising from multiple comparisons, FDR values were computed using the Benjamini–Hochberg correction method, which adjusts raw *p*-values to limit the expected proportion of false discoveries. From the resulting list, the top five most relevant pathways from each data source (GO Biological Processes, Reactome, and Monarch) were selected based on their established association with neuropsychiatric disorder-related biological processes and phenotypic manifestations. This prioritized subset enabled systematic comparison of the natural compounds’ potential mechanistic impacts on neuropsychiatric pathophysiology.

The prioritized Gene Ontology (GO) Biological Processes included (1) ‘Cognition’, (2) ‘Regulation of synaptic transmission, glutamatergic’, (3) ‘Nervous system development’, (4) ‘Learning or memory’, and (5) ‘Regulation of neurogenesis’—all critically involved in synaptic plasticity and neural circuit function. From Reactome, the selected pathways comprised (1) ‘Axon guidance’, (2) ‘PIP3 activates AKT signaling’, (3) ‘Signaling by NTRKs’, (4) ‘Transmission across Chemical Synapses’, and (5) ‘Neuronal system’, representing core mechanisms of neuronal development and communication. The Monarch phenotypes—(1) ‘Autistic behavior’, (2) ‘Neurodevelopmental abnormality’, (3) ‘Neurodevelopmental delay’, (4) ‘Intellectual disability’, and (5) ‘Cognitive impairment’—were chosen as they capture the essential clinical manifestations of neurodevelopmental disorders.

### 4.7. Construction of the Compound–Target–Pathway Network

An integrated network was constructed using Cytoscape 3.10.3 to visualize the complex interactions between the natural compounds, their molecular targets, and the selected neurodevelopmental pathways. Network topology analysis was performed using the CytoHubba 0.1 plugin, where targets were ranked by degree centrality to identify the most functionally significant nodes within the network architecture. This approach enabled the systematic identification of hub targets based on their connectivity patterns and presumed biological importance in the context of neurodevelopmental processes.

The three most highly connected targets, identified based on their degree values within the network, were selected for investigation through molecular docking simulations. This enabled the evaluation of potential interactions between the natural compounds and these prioritized targets.

### 4.8. Molecular Docking

The molecular docking analysis was performed to characterize binding interactions between the natural compounds and prioritized targets. Three-dimensional protein structures were obtained from the Protein Data Bank (https://www.rcsb.org/) and prepared using Discovery Studio 2021, involving (1) the removal of crystallographic water molecules and non-essential ligands, (2) the addition of polar hydrogen atoms, and (3) the definition of binding site grid parameters. Protein structures underwent energy minimization using Swiss-PDB Viewer to optimize molecular geometry prior to docking simulations.

The 3D structures of the selected natural compounds were obtained from the PubChem database (https://pubchem.ncbi.nlm.nih.gov/) and converted to PDB format using Open Babel 3.1.1 software. Molecular docking simulations were performed using AutoDock Vina 1.1.2 [70], with a defined grid box of 15 × 15 × 15 Å centered on the crystallographic coordinates of each protein’s binding site. 

The most stable binding conformations were selected based on root-mean-square deviation (RMSD) values ≤ 2.0 Å, ensuring structural similarity and conformational stability of the docked poses. Molecular interactions were systematically characterized using Discovery Studio 2021, applying established criteria: (1) hydrogen bonds < 3.3 Å [71] and (2) hydrophobic interactions including π-π, π-alkyl, and π-sigma contacts < 6.0 Å [72].

### 4.9. Data Analysis and Graphical Visualization in RStudio

The ggplot2 package was implemented to generate comprehensive visualizations, including (1) number line plots displaying predicted LC50 values, (2) boxplots illustrating quartile-based toxicity classification, (3) scatter plots of biological activity probabilities, (4) enriched pathway representations, and (5) heatmaps of molecular docking results. Toxicity probability quartiles were calculated using the dplyr::ntile(row_number(), (4) function, ensuring systematic compound stratification. Target overlap between natural compounds and neurodevelopmental disorders (NDDs) was visualized using Euler diagrams constructed with the eulerr package.

## 5. Conclusions

This study highlights the promising neuroprotective potential of the natural compounds caryophyllene oxide (BCPO), linoleic acid (LA), and tangeretin (TG) through their multi-target interactions with key proteins (CSNK2B, GRIN1, and MAPK1) and modulation of neurodevelopmental pathways. Computational analyses revealed favorable binding affinities, BBB permeability, and low toxicity profiles, positioning these compounds as viable candidates for further investigation in neurodevelopmental and neurodegenerative disorders.

Future preclinical studies should validate these findings through in vitro and in vivo experiments to assess their efficacy, optimal dosing, and safety, paving the way for potential therapeutic applications. The integration of computational and experimental approaches presented here provides a robust framework for developing novel, multi-target neuroprotective agents.

## Figures and Tables

**Table 1:** The pharmacological parameters of the selected natural compounds obtained from the TCMSP database and the SwissADME platform.

	TCMSP					SwissADME			
Compound	Molecular Weight	OB (%)	CaCo-2	BBB	DL	GI Absorption	BBB Permeant	Bioavailability Score	Lipinski
Kobusone	222.36	39.16	1.01	1.08	0.13	High	Yes	0.55	Yes
Miltionone II	312.39	71.03	0.62	0.03	0.44	High	Yes	0.85	Yes
Caryophyllene oxide	220.39	34.43	1.09	1.81	0.13	High	Yes	0.55	Yes
α-humulene epoxide	220.39	23.66	1.58	1.77	0.10	High	Yes	0.55	Yes
Selina-4(14),7(11)-dien-8-one	218.37	32.31	1.42	0.57	0.10	High	Yes	0.55	Yes
Osthol	244.31	38.75	1.15	0.85	0.13	High	Yes	0.55	Yes
Isokobusone	222.36	39.63	0.67	0.51	0.11	High	Yes	0.55	Yes
Linoleic acid	280.50	41.90	1.16	0.90	0.14	High	Yes	0.85	Yes
Tangeretin	372.40	21.38	1.23	0.09	0.43	High	Yes	0.55	Yes
Sinensetin	372.40	50.56	1.12	0.04	0.45	High	Yes	0.55	Yes

OB = oral bioavailability, CaCo-2 = intestinal permeability, BBB = blood–brain barrier permeability, GI = gastrointestinal absorption, and DL = drug-likeness.

## Data Availability

The original contributions presented in this study are included in the article/Supplementary Material. Further inquiries can be directed to the corresponding author.

## Supplementary Materials

The following supporting information can be downloaded at https://www.mdpi.com/article/10.3390/ijms26188873/s1.

## References

[1] Homberg J.R., Kyzar E.J., Scattoni M.L., Norton W.H., Pittman J., Gaikwad S., Nguyen M., Poudel M.K., Ullmann J.F.P., Diamond D.M. (2016). Genetic and Environmental Modulation of Neurodevelopmental Disorders: Translational Insights from Labs to Beds. Brain Res. Bull..

[2] Thapar A., Cooper M., Rutter M. (2017). Neurodevelopmental Disorders. Lancet Psychiatry.

[3] American Psychiatric Association (2013). Diagnostic and Statistical Manual of Mental Disorders.

[4] Ijomone O.M., Olung N.F., Akingbade G.T., Okoh C.O.A., Aschner M. (2020). Environmental Influence on Neurodevelopmental Disorders: Potential Association of Heavy Metal Exposure and Autism. J. Trace Elem. Med. Biol..

[5] Parenti I., Rabaneda L.G., Schoen H., Novarino G. (2020). Neurodevelopmental Disorders: From Genetics to Functional Pathways. Trends Neurosci..

[6] Wozniak R.H., Leezenbaum N.B., Northrup J.B., West K.L., Iverson J.M. (2017). The Development of Autism Spectrum Disorders: Variability and Causal Complexity. WIREs Cogn. Sci..

[7] Doi M., Usui N., Shimada S. (2022). Prenatal Environment and Neurodevelopmental Disorders. Front. Endocrinol..

[8] Ozlu C., Bailey R.M., Sinnett S., Goodspeed K.D. (2021). Gene Transfer Therapy for Neurodevelopmental Disorders. Dev. Neurosci..

[9] Ghiani C.A., Faundez V. (2017). Cellular and Molecular Mechanisms of Neurodevelopmental Disorders. J. Neurosci. Res..

[10] Jiang C.-C., Lin L.-S., Long S., Ke X.-Y., Fukunaga K., Lu Y.-M., Han F. (2022). Signalling Pathways in Autism Spectrum Disorder: Mechanisms and Therapeutic Implications. Signal Transduct. Target. Ther..

[11] Baranova J., Dragunas G., Botellho M.C.S., Ayub A.L.P., Bueno-Alves R., Alencar R.R., Papaiz D.D., Sogayar M.C., Ulrich H., Correa R.G. (2021). Autism Spectrum Disorder: Signaling Pathways and Prospective Therapeutic Targets. Cell Mol. Neurobiol..

[12] Sadybekov A.V., Katritch V. (2023). Computational Approaches Streamlining Drug Discovery. Nature.

[13] Shaker B., Ahmad S., Lee J., Jung C., Na D. (2021). In Silico Methods and Tools for Drug Discovery. Comput. Biol. Med..

[14] Xia S., Chen E., Zhang Y. (2023). Integrated Molecular Modeling and Machine Learning for Drug Design. J. Chem. Theory Comput..

[15] Bassani D., Moro S. (2023). Past, Present, and Future Perspectives on Computer-Aided Drug Design Methodologies. Molecules.

[16] Tang Q., Chu J., Peng P., Zou Y., Wu Y., Wang Y. (2025). Probing the Antibacterial Mechanism of Aloe Vera Based on Network Pharmacology and Computational Analysis. J. Mol. Graph. Model..

[17] Hopkins A.L. (2008). Network Pharmacology: The next Paradigm in Drug Discovery. Nat. Chem. Biol..

[18] Hopkins A.L. (2007). Network Pharmacology. Nat. Biotechnol..

[19] Liu Y., Li H., Wang X., Huang J., Zhao D., Tan Y., Zhang Z., Zhang Z., Zhu L., Wu B. (2023). Anti-Alzheimers Molecular Mechanism of Icariin: Insights from Gut Microbiota, Metabolomics, and Network Pharmacology. J. Transl. Med..

[20] Niu B., Xie X., Xiong X., Jiang J. (2022). Network Pharmacology-Based Analysis of the Anti-Hyperglycemic Active Ingredients of Roselle and Experimental Validation. Comput. Biol. Med..

[21] Yu X., Qin W., Cai H., Ren C., Huang S., Lin X., Tang L., Shan Z., AL-Ameer W.H.A., Wang L. (2024). Analyzing the Molecular Mechanism of Xuefuzhuyu Decoction in the Treatment of Pulmonary Hypertension with Network Pharmacology and Bioinformatics and Verifying Molecular Docking. Comput. Biol. Med..

[22] Zhang Y., Li Z., Wei J., Kong L., Song M., Zhang Y., Xiao X., Cao H., Jin Y. (2022). Network Pharmacology and Molecular Docking Reveal the Mechanism of Angelica Dahurica against Osteosarcoma. Medicine.

[23] Asgharian P., Quispe C., Herrera-Bravo J., Sabernavaei M., Hosseini K., Forouhandeh H., Ebrahimi T., Sharafi-Badr P., Tarhriz V., Soofiyani S.R. (2022). Pharmacological Effects and Therapeutic Potential of Natural Compounds in Neuropsychiatric Disorders: An Update. Front. Pharmacol..

[24] Ashmawy N.S., Gad H.A., El-Nashar H.A.S. (2023). Comparative Study of Essential Oils from Different Organs of Syzygium Cumini (Pamposia) Based on GC/MS Chemical Profiling and In Vitro Antiaging Activity. Molecules.

[25] Grosso C., Santos M., Barroso M.F. (2023). From Plants to Psycho-Neurology: Unravelling the Therapeutic Benefits of Bioactive Compounds in Brain Disorders. Antioxidants.

[26] Bhandari R., Kuhad A. (2015). Neuropsychopharmacotherapeutic Efficacy of Curcumin in Experimental Paradigm of Autism Spectrum Disorders. Life Sci..

[27] Sachdeva P., Mehdi I., Kaith R., Ahmad F., Anwar M.S. (2022). Potential Natural Products for the Management of Autism Spectrum Disorder. Ibrain.

[28] Corona J.C. (2018). Natural Compounds for the Management of Parkinson’s Disease and Attention-Deficit/Hyperactivity Disorder. Biomed. Res. Int..

[29] Ballardin D., Cruz-Gamero J.M., Bienvenu T., Rebholz H. (2022). Comparing Two Neurodevelopmental Disorders Linked to CK2: Okur-Chung Neurodevelopmental Syndrome and Poirier-Bienvenu Neurodevelopmental Syndrome—Two Sides of the Same Coin?. Front. Mol. Biosci..

[30] Borgo C., D’Amore C., Sarno S., Salvi M., Ruzzene M. (2021). Protein Kinase CK2: A Potential Therapeutic Target for Diverse Human Diseases. Signal Transduct. Target. Ther..

[31] Liu Y., Xia D., Zhong L., Chen L., Zhang L., Ai M., Mei R., Pang R. (2024). Casein Kinase 2 Affects Epilepsy by Regulating Ion Channels: A Potential Mechanism. CNS Neurol. Disord. Drug Targets.

[32] Canedo-Antelo M., Serrano M.P., Manterola A., Ruiz A., Llavero F., Mato S., Zugaza J.L., Pérez-Cerdá F., Matute C., Sánchez-Gómez M.V. (2018). Inhibition of Casein Kinase 2 Protects Oligodendrocytes from Excitotoxicity by Attenuating JNK/P53 Signaling Cascade. Front. Mol. Neurosci..

[33] Marshall C.A., McBride J.D., Changolkar L., Riddle D.M., Trojanowski J.Q., Lee V.M.-Y. (2022). Inhibition of CK2 Mitigates Alzheimer’s Tau Pathology by Preventing NR2B Synaptic Mislocalization. Acta Neuropathol. Commun..

[34] Castello J., Ragnauth A., Friedman E., Rebholz H. (2017). CK2—An Emerging Target for Neurological and Psychiatric Disorders. Pharmaceuticals.

[35] Korinek M., Candelas Serra M., Abdel Rahman F.E.S., Dobrovolski M., Kuchtiak V., Abramova V., Fili K., Tomovic E., Hrcka Krausova B., Krusek J. (2024). Disease-Associated Variants in GRIN1, GRIN2A and GRIN2B Genes: Insights into NMDA Receptor Structure, Function, and Pathophysiology. Physiol. Res..

[36] Mony L., Paoletti P. (2023). Mechanisms of NMDA Receptor Regulation. Curr. Opin. Neurobiol..

[37] Rouzbeh N., Rau A.R., Benton A.J., Yi F., Anderson C.M., Johns M.R., Jensen L., Lotti J.S., Holley D.C., Hansen K.B. (2023). Allosteric Modulation of GluN1/GluN3 NMDA Receptors by GluN1-Selective Competitive Antagonists. J. Gen. Physiol..

[38] Xu Y., Song R., Chen W., Strong K., Shrey D., Gedela S., Traynelis S.F., Zhang G., Yuan H. (2021). Recurrent Seizure-related GRIN1 Variant: Molecular Mechanism and Targeted Therapy. Ann. Clin. Transl. Neurol..

[39] He M., Wollmuth L.P. (2023). Activation of Excitatory Glycine NMDA Receptors: At the Mercy of a Whimsical GluN1 Subunit. J. Gen. Physiol..

[40] Urdaneta K.E., Castillo M.A., Montiel N., Semprún-Hernández N., Antonucci N., Siniscalco D. (2018). Autism Spectrum Disorders: Potential Neuro-Psychopharmacotherapeutic Plant-Based Drugs. Assay Drug Dev. Technol..

[41] Gyrdymova Y.V., Rubtsova S.A. (2022). Caryophyllene and Caryophyllene Oxide: A Variety of Chemical Transformations and Biological Activities. Chem. Pap..

[42] Moghrovyan A., Parseghyan L., Sevoyan G., Darbinyan A., Sahakyan N., Gaboyan M., Karabekian Z., Voskanyan A. (2022). Antinociceptive, Anti-Inflammatory, and Cytotoxic Properties of Origanum Vulgare Essential Oil, Rich with β-Caryophyllene and β-Caryophyllene Oxide. Korean J. Pain.

[43] Karakaya S., Yilmaz S.V., Özdemir Ö., Koca M., Pınar N.M., Demirci B., Yıldırım K., Sytar O., Turkez H., Baser K.H.C. (2020). A Caryophyllene Oxide and Other Potential Anticholinesterase and Anticancer Agent in Salvia Verticillata Subsp. Amasiaca (Freyn & Bornm.) Bornm. (Lamiaceae). J. Essent. Oil Res..

[44] Dougnon G., Ito M. (2021). Essential Oil from the Leaves of Chromolaena Odorata, and Sesquiterpene Caryophyllene Oxide Induce Sedative Activity in Mice. Pharmaceuticals.

[45] Oppong-Damoah A., Blough B.E., Makriyannis A., Murnane K.S. (2019). The Sesquiterpene Beta-Caryophyllene Oxide Attenuates Ethanol Drinking and Place Conditioning in Mice. Heliyon.

[46] Whelan J., Fritsche K. (2013). Linoleic Acid. Adv. Nutr..

[47] Djuricic I., Calder P.C. (2021). Beneficial Outcomes of Omega-6 and Omega-3 Polyunsaturated Fatty Acids on Human Health: An Update for 2021. Nutrients.

[48] Alarcon-Gil J., Sierra-Magro A., Morales-Garcia J.A., Sanz-SanCristobal M., Alonso-Gil S., Cortes-Canteli M., Niso-Santano M., Martínez-Chacón G., Fuentes J.M., Santos A. (2022). Neuroprotective and Anti-Inflammatory Effects of Linoleic Acid in Models of Parkinson’s Disease: The Implication of Lipid Droplets and Lipophagy. Cells.

[49] Mbiydzenyuy N.E., Ninsiima H.I., Valladares M.B., Pieme C.A. (2018). Zinc and Linoleic Acid Pre-Treatment Attenuates Biochemical and Histological Changes in the Midbrain of Rats with Rotenone-Induced Parkinsonism. BMC Neurosci..

[50] Kousparou C., Fyrilla M., Stephanou A., Patrikios I. (2023). DHA/EPA (Omega-3) and LA/GLA (Omega-6) as Bioactive Molecules in Neurodegenerative Diseases. Int. J. Mol. Sci..

[51] Taha A.Y. (2020). Linoleic Acid–Good or Bad for the Brain?. NPJ Sci. Food.

[52] Dec K., Alsaqati M., Morgan J., Deshpande S., Wood J., Hall J., Harwood A.J. (2023). A High Ratio of Linoleic Acid (n-6 PUFA) to Alpha-Linolenic Acid (n-3 PUFA) Adversely Affects Early Stage of Human Neuronal Differentiation and Electrophysiological Activity of Glutamatergic Neurons in Vitro. Front. Cell Dev. Biol..

[53] Fatima J., Siddique Y.H. (2025). The Neuroprotective Role of Tangeritin. CNS Neurol. Disord. Drug Targets.

[54] de Luna F.C.F., Ferreira W.A.S., Casseb S.M.M., de Oliveira E.H.C. (2023). Anticancer Potential of Flavonoids: An Overview with an Emphasis on Tangeretin. Pharmaceuticals.

[55] Wani I., Koppula S., Balda A., Thekkekkara D., Jamadagni A., Walse P., Manjula S.N., Kopalli S.R. (2024). An Update on the Potential of Tangeretin in the Management of Neuroinflammation-Mediated Neurodegenerative Disorders. Life.

[56] Datla K.P., Christidou M., Widmer W.W., Rooprai H.K., Dexter D.T. (2001). Tissue Distribution and Neuroprotective Effects of Citrus Flavonoid Tangeretin in a Rat Model of Parkinson’s Disease. Neuroreport.

[57] Alla N., Palatheeya S., Challa S.R., Kakarla R. (2024). Tangeretin Confers Neuroprotection, Cognitive and Memory Enhancement in Global Cerebral Ischemia in Rats. 3 Biotech.

[58] Arafa E.-S.A., Shurrab N.T., Buabeid M.A. (2021). Therapeutic Implications of a Polymethoxylated Flavone, Tangeretin, in the Management of Cancer via Modulation of Different Molecular Pathways. Adv. Pharmacol. Pharm. Sci..

[59] Posit team (2024). RStudio: Integrated Development Environment for R.

[60] Goel R.K., Singh D., Lagunin A., Poroikov V. (2011). PASS-Assisted Exploration of New Therapeutic Potential of Natural Products. Med. Chem. Res..

[61] Bagli E., Goussia A., Moschos M.M., Agnantis N., Kitsos G. (2016). Natural Compounds and Neuroprotection: Mechanisms of Action and Novel Delivery Systems. Vivo.

[62] Mohd Sairazi N.S., Sirajudeen K.N.S. (2020). Natural Products and Their Bioactive Compounds: Neuroprotective Potentials against Neurodegenerative Diseases. Evid. Based Complement. Altern. Med..

[63] Trott O., Olson A.J. (2010). AutoDock Vina: Improving the Speed and Accuracy of Docking with a New Scoring Function, Efficient Optimization, and Multithreading. J. Comput. Chem..

[64] McREE D.E., McREE D.E. (1999). Computational Techniques. Practical Protein Crystallography.

[65] Ribas J., Cubero E., Luque F.J., Orozco M. (2002). Theoretical Study of Alkyl-π and Aryl-π Interactions. Reconciling Theory and Experiment. J. Org. Chem..

[66] Niazi S.K., Mariam Z. (2023). Computer-Aided Drug Design and Drug Discovery: A Prospective Analysis. Pharmaceuticals.

[67] Salmanli M., Tatar Yilmaz G., Tuzuner T. (2021). Investigation of the Antimicrobial Activities of Various Antimicrobial Agents on Streptococcus Mutans Sortase A through Computer-Aided Drug Design (CADD) Approaches. Comput. Methods Programs Biomed..

[68] Correia A.C., Monteiro A.R., Silva R., Moreira J.N., Sousa Lobo J.M., Silva A.C. (2022). Lipid Nanoparticles Strategies to Modify Pharmacokinetics of Central Nervous System Targeting Drugs: Crossing or Circumventing the Blood–Brain Barrier (BBB) to Manage Neurological Disorders. Adv. Drug Deliv. Rev..

[69] Wu F., Zhou Y., Li L., Shen X., Chen G., Wang X., Liang X., Tan M., Huang Z. (2020). Computational Approaches in Preclinical Studies on Drug Discovery and Development. Front. Chem..

[70] Wang Y., Hu B., Feng S., Wang J., Zhang F. (2020). Target Recognition and Network Pharmacology for Revealing Anti-Diabetes Mechanisms of Natural Product. J. Comput. Sci..

[71] Li J., Gao K., Cai S., Liu Y., Wang Y., Huang S., Zha J., Hu W., Yu S., Yang Z. (2019). Germline de Novo Variants in CSNK2B in Chinese Patients with Epilepsy. Sci. Rep..

[72] Al-Ayadhi L., Bhat R.S., Alghamdi F.A., Alhadlaq A.S., El-Ansary A. (2023). Influence of Auditory Integrative Training on Casein Kinase 2 and Its Impact on Behavioral and Social Interaction in Children with Autism Spectrum Disorder. Curr. Issues Mol. Biol..

